# Isolation and characterization of biosurfactant-producing microbes isolated from the gastrointestinal system of broiler birds fed a commercial diet

**DOI:** 10.1080/10495398.2023.2263771

**Published:** 2023-10-10

**Authors:** Ngavaite Chigede, Zedias Chikwambi, Irvin D. T. Mpofu, James Madzimure

**Affiliations:** a School of Agricultural Sciences and Technology, Department of Animal Production and Technology, Chinhoyi University of Technology, Chinhoyi, Zimbabwe; b Gary Magadzire School of Agriculture and Engineering, Department of Livestock, Wildlife and Fisheries, Great Zimbabwe University, Masvingo, Zimbabwe; c College of Health, Agriculture and Natural Sciences, Department of Agricultural Sciences, Africa University, Mutare, Zimbabwe

**Keywords:** Antimicrobial, beta lactams, decane, emulsification index, endogenous biosurfactants, haemolytic activity, 16S rRNA

## Abstract

Antimicrobial drug resistance (AMR) from improper use of antibiotics in various livestock products is a growing hazard for humans worldwide, with current death rate in excess of 700,000 per annum linked to the problem. Microorganisms are a rich source of structurally distinct bioactive compounds designed to protect the microbes and can offset AMR challenge. A study was conducted at Chinhoyi University of Technology to isolate, identify and characterize biosurfactant secreting microbes from broiler bird’s gastrointestinal tract. Analysis of variance was performed in Genstat software. 16S rRNA technique was used to identify the DNA of isolates, annotated by similarity using BLASTn analysis against the NCBI nucleotide database. Phylogenetic analysis was performed on the BLASTn outcome to have an appreciation of the evolutionary genetic relationships. Small intestine-derived samples had a wider hemolytic activity of 5.6 mm, with a 39% emulsification index. At 98.29% sequence similarity, the bacterium producing biosurfactants was identified as an *Escherichia coli* strain similar to the 7.1994/NIST 0056 strain. The biosurfactant substance is a derivative of decane with beta lactams, tetracyclines and sulfa drugs properties which were responsible for the observed antibacterial activity. We recommend endogenous biosurfactant production optimization experiments and *in-vivo* trials to evaluate the potential impacts of a biosurfactant based feed additive in broilers.

## Introduction

Natural products are regarded as important compounds which exhibit many applications in the field of agriculture among others.^1^ Veterinary medications are widely used in the production of food animals for therapeutic and preventative purposes. If these medications are misused or the advised drug withdrawal times are not followed, residues of these compounds may remain in the animal food products, and the risk they pose to human health cannot be disregarded.^2^ It is therefore of paramount importance to advocate for products which pose less risk to human health than synthetic products.

Biosurfactants are amphipathic molecules with a wide range of structural variations, biodegradability and less toxicity compared to their synthetic counterparts.^1^ Several biosurfactants capabilities include antibacterial, antifungal, and antiviral properties, making them useful molecules in the fight against a variety of diseases and infections.^3–5^ Biosurfactants can also be used as anti-adhesive agents against infections. In this way, the release of biosurfactants by probiotic bacteria *in-vivo* is a defence weapon against other colonization strains in the gastrointestinal tracts.^5^^,^^6^ Microbes can survive a wide range of stressful situations thanks to their diverse biosurfactant properties, allowing them to conquer a wide range of settings. These functions can be exploited and used to alter the productivity of broiler chickens.

The demand for developing clean, nontoxic, and environmentally friendly synthetic approaches (green chemistry) in the synthesis of various agricultural compounds that will end up in the environment has increased in response to the world’s growing environmental concerns.^7^ The creation of advanced bio-based materials is the result of this. Synthetic surfactants can be replaced with microbial surfactants (biosurfactants), which also have potential applications in the biomedical, industrial, and environmental fields as well as being potential antibiotic agents.^1^ Low toxicity, improved environmental compatibility, high selectivity, and specific activity at extremely adverse conditions like pH, temperature, and salinity are just a few of the intriguing characteristics of biosurfactants.^7^ These factors make biosurfactants potential candidates for a variety of uses, such as their use in the field of animal sciences. Depending on the microorganisms that produce them, various types of biosurfactants exhibit a wide range of physiological functions and have different properties.^8^ Hydrophobic compound solubilization, heavy metal binding, virulence factors, cell signaling (quorum sensing), and biofilm formation are noteworthy among all of these characteristics. However, an accurate characterization of the compounds and any potential toxic side effects is necessary for their potential application in a variety of products, such as feed additives. This study presents isolation, identification and characterization of endogenous biosurfactants secreted by a gut extracted microbe in broilers fed a commercial diet with the objective of counteracting antimicrobial resistance challenge.

## Materials and methods

### Study site

The study was conducted at Zimbabwe’s Chinhoyi University of Technology (CUT) biotechnology laboratory located 17.3533° S and 30.2058° E. CUT farm bred broiler chickens utilizing a deep litter system with wheat straw as bedding were used. The farm is situated at an altitude of 1140 meters in a sub-humid tropical setting. The average annual rainfall at the farm site is 850 mm, and the average daily temperature ranges from 7 °C in winter to 27 °C in summer. Cambisols which are granite-derived soils are present at the farm.^9^

### Sample collection

The birds, from which samples were taken, were taken care of in accordance with the established regulations in ‘The governance of animal care and use for scientific purposes in Africa and the Middle East’.^10^ Birds were kept under similar conditions to those under which commercial farm animals are kept hence authorization from an Ethics Committee were not necessary as per directive no. 2010/63/EU of the European Parliament and of the Council.

The gastrointestinal tract (GIT) of healthy chickens which were fed a commercial diet was collected at slaughter age of six weeks and transported on ice to the laboratory. The GIT was sectioned into four tissues: crop, gizzard, small and large intestines. The digesta samples from each tissue section were then aseptically collected into 2 mL Eppendorf tubes for immediate culturing. Collected GIT samples were serially diluted (up to 10^−5^) in 0.85% ringer solution. Serial dilutions of each region were spread plated in triplicate on nonselective multi-nutrient agar medium (mass/volume: 0.5% peptone, 0.3% beef extract, 1.5% agar) and incubated at 30 °C for 24 h. The plate count method, as published by the,^11^ was used to determine the microbial population. After the incubation phase, colony forming units (cfu) were determined using a colony counter.

### Isolation of potential biosurfactant producing bacteria

The hemolytic activity, oil spread, and oil drop collapse assays were used to identify potential biosurfactant secreting bacteria.

#### Hemolytic activity

The method described by Walter et al.^12^ was followed. Blood agar base was prepared according to the manufacturer’s directions, then sterile sheep blood at a rate of 5% v/v was added at a temperature of 55 °C in a water bath. The liquid mixture was gently stirred until the blood was evenly dispersed, and then it was poured aseptically into petri plates. Sheep blood was used in the blood agar because of its’ increased sensitivity to the hemolytic toxins released by bacterial cells thus causing hemolytic zones around the colonies over the period of time.^13^

Bacterial colonies which were grown on nonselective multi-nutrient agar medium were transferred to 5% (v/v) sheep blood agar using a sterile Whatman filter paper size 1 (in place of velveteen membrane). First, the Whatman filter paper was cut into circular disks which can fit inside a petri dish with a flip of paper on one end to facilitate easy lifting of the paper. The cut Whatman filter papers were then steam autoclaved for sterilization purposes. Colony lifting was done aseptically in a lamina flow. A velveteen membrane was carefully placed on top of nutrient agar plates that had been incubated at 30 °C for 24 h. The membrane copied microbial cells which were then transferred onto blood agar plates, where they were gently pushed to imprint the cells onto the blood agar. For 48 h, the cultures were incubated at 30 °C. The appearance of a clear zone surrounding the bacterial colonies indicated hemolytic action.^13^ After testing for hemolytic activity, colonies with clear zones on blood agar were streaked and subcultured on multi-nutrient agar. This was done to ensure that the cultures were pure.

Following the hemolysis test, pure colonies were transferred to a multi-nutrient broth medium and cultured for six days at 30 °C. Biosurfactants were extracted from supernatant by centrifugation at 5 000 rpm for 20 min. The supernatant was collected for additional screening tests, that is the oil spread method, oil drop collapse, and emulsification activity.^14^

#### Oil spreading method

Distilled water (40 mL) was poured on to the petri dishes, followed by addition of vegetable oil (10 µL) placed to the center of the petri dish. Following that, a drop of cell-free culture broth supernatant (10 µL) was put over the vegetable oil surface. The diameter of the clearance zone on the oil surface was measured and compared to that of the negative control (10 µL of distilled water).^14^

#### Oil drop collapse method

The method described by Jain et al.^15^ was followed. In each petri plate, ten microlitres of vegetable oil were put. After that, 10 µL of cell-free culture broth were added, and the drop on the oil surface image was examined after 2 m. When the cultures produced a flat drop, this was deemed positive biosurfactant production. Isolates that produced round droplets were scored as negative, indicating a lack of biosurfactant production.^12^

### Complementary screening of biosurfactant producing bacteria

Microbial isolates that were positive for at least one primary screening method were subjected to an emulsification capacity assay as a supplement to confirm their potential to secrete biosurfactants. An emulsification index (E_24_) devised by Cooper and Goldenberg^16^ was used to assess the emulsifying potential of isolated strains. Six milliliters of vegetable oil were mixed with four milliliters of culture supernatant. For 2 m, the liquid was vortexed at high speed to fully combine the supernatant and oil. The combination was left to stand for 24 h. The E_24_ index was derived by dividing the height of the emulsified layer (mm) by the overall height of the liquid column (mm) (i.e., height of oil + emulsion layer).^12^^,^^17^ The results were compared to distilled water, which served as a negative control.^14^

### Identification of biosurfactant secreting bacterial isolates

A method described by Rayeni and Nezhad^14^ was followed. Ten milliliters of an overnight culture of selected microbe, in multi-nutrient broth, was added to 500 mL of multi-nutrient broth and incubated for 7 days at 30 °C. To recover the biosurfactant, the bacteria were eliminated by centrifuging at 5000 rpm for 20 m. The pH of the supernatant was adjusted to 2 with 6 N HCI and then the solution was stored at 4 °C for 24 h to precipitate the biosurfactants. The biosurfactant within the organic layer were obtained by vigorously mixing a solution of chloroform and methanol (2:1 v/v) with the precipitated biosurfactants. Solvents were opted for due to their extensive capacity to solubilize a number of secondary metabolites. Also, methanol contains both polar and nonpolar groups which make it able to extract both polar and nonpolar compounds. Mixing ensures movement of biosurfactants from the hydrophilic phase (nutrient broth) into the organic, hydrophobic phase.^14^ This layer was separated using a separating funnel and dried at 50 °C for 4–5 h to obtain dry mass which was then taken for further analysis using a GC/MS and FTIR spectroscopy to determine chemical components. The biosurfactant samples were first screened for antibiotic properties using radio receptor assay at the Residue Analysis Division of the Central Veterinary Laboratory (CVL), Harare. The assay detects substances in the sample that have characteristics similar to a particular antibiotic drug. Biosurfactants were then evaluated for their *in-vitro* antibacterial properties against *Escherichia coli* and *Staphylococcus aureus* using the agar well diffusion method.^18^ Studies were conducted using distilled water as a negative control and Terranox (positive control), which contained oxytetracycline soluble powder at a recommended rate of 2 mg per 1 mL of water. The zones of growth inhibition (mm) around the disks were measured after 24 h of incubation at 37 °C. Obtained data were analyzed using a one-way analysis of variance (ANOVA) in Genstat 18th edition and means were separated using Fischer’s least significant difference at 5% confidence interval.

Isolates positive for the biosurfactant production screening tests were taken for identification using the 16S rRNA technique. Bacterial DNA was extracted from the culture using the Quick-DNA™ Fungal/Bacterial Minirep Kit (ZYMO RESEARCH, Catalogue No. D6005) according to the manufacturer’s protocol. The concentration and purity of the extracted DNA was determined using an absorption spectroscopy model at wavelengths 260 nm and 280 nm. The integrity of the PCR amplicons was visualized on 1% agarose gel (CSL-AG500, Cleaver Scientific Ltd) stained with EZ-vision^®^ Bluelight DNA Dye. The NEB Fast Ladder was used on all gels (N3238) as size standard. The 16S gene target region (27–1492 bp) was amplified using the universal primer sets from Inqaba Biotechnology, South Africa: 16S-27F5′ – AGAGTTTGATCMTGGCTCAG −3′ and 16S-1492R 5′ – CGGTTACCTTGTTACGACTT −3′.^19^ The PCR was realized on a thermal cycler under the following conditions: NEB OneTaq 2x MasterMix with standard buffer (Catalogue No. M0482S), Genomic DNA (10–30 ng/µl), Forward primer (10 µM), Reverse primer (10 µM), and Nuclease free water (Catalogue No. E476). Amplification was performed using the initial denaturation at 94 °C for 5 m, followed by 35 cycles of denaturation at 94 °C for 30 s, annealing at 50 °C for 30 s, extension at 68 °C for 1 m, and a final extension at 68 °C for 10 m.

The amplicons were enzymatically purified using the ExoSAP procedure (NEB M0293L; NEB M0371), for sequencing (zymo Research, ZR-96 DNA Sequencing Clean-up Kit™, Catalogue No. D4050), and sequenced in the forward and reverse direction (Nimagen, BrilliantDye™ Terminator cycle sequencing Kit V3.1, BRD3-100/1000) using the ABI 3730x/Genetic analyzer (Applied Biosystems, Thermo Fisher Scientific).

FinchTV (https://finchtv.software.informer.com/1.4/) was used to view the raw chromatogram files (.abi). CLC Bio Main Workbench was used to assemble the forward and reverse sequencing reads to form a consensus sequence for each sample. BLASTn analysis (with default parameters) was performed against NCBI website (https://blast.ncbi.nlm.nih.gov/Blast.cgi) to determine if a sequence in the database matches the query sequence above a certain threshold (99% query coverage; 99% identity).

Similarity scores from BLASTn analysis were extracted into a binary table (https://doi.org/10.6084/m9.figshare.23574249.v1) for phylogenetic diversity analysis between the identified strains of bacterial species. Phylogenetic analysis was done using the Unweighted Pair Group Method (UPGM) of cluster analysis in MultiVariate Statistical Package (MVSP) version 3.22 Kovach Computing Services.

## Results

### Broiler birds GIT microbial distribution map

There were variations in microbial populations along the gastrointestinal tract of birds, with significant differences between the proximal gut compartments (crop and gizzard) and distal compartments (intestines) (Table 1).

**Table 1.** Average colony forming units per milliliter for each GIT section.

GIT section	Mean cfu/mL
Crop	1.E + 10^a^
Gizzard	1.E + 10^a^
Small intestines	1.E + 09^b^
Large intestines	5.E + 09^c^
Grand mean	8.E + 09
*p* Value	<0.05

Rows with the same superscript are statistically insignificant while a different superscript means statistically different means at 5% significance level.

The crop and gizzard had the same microbial populations which were different from those from the small and large intestines.

### Hemolytic activity

**Figure 1.**
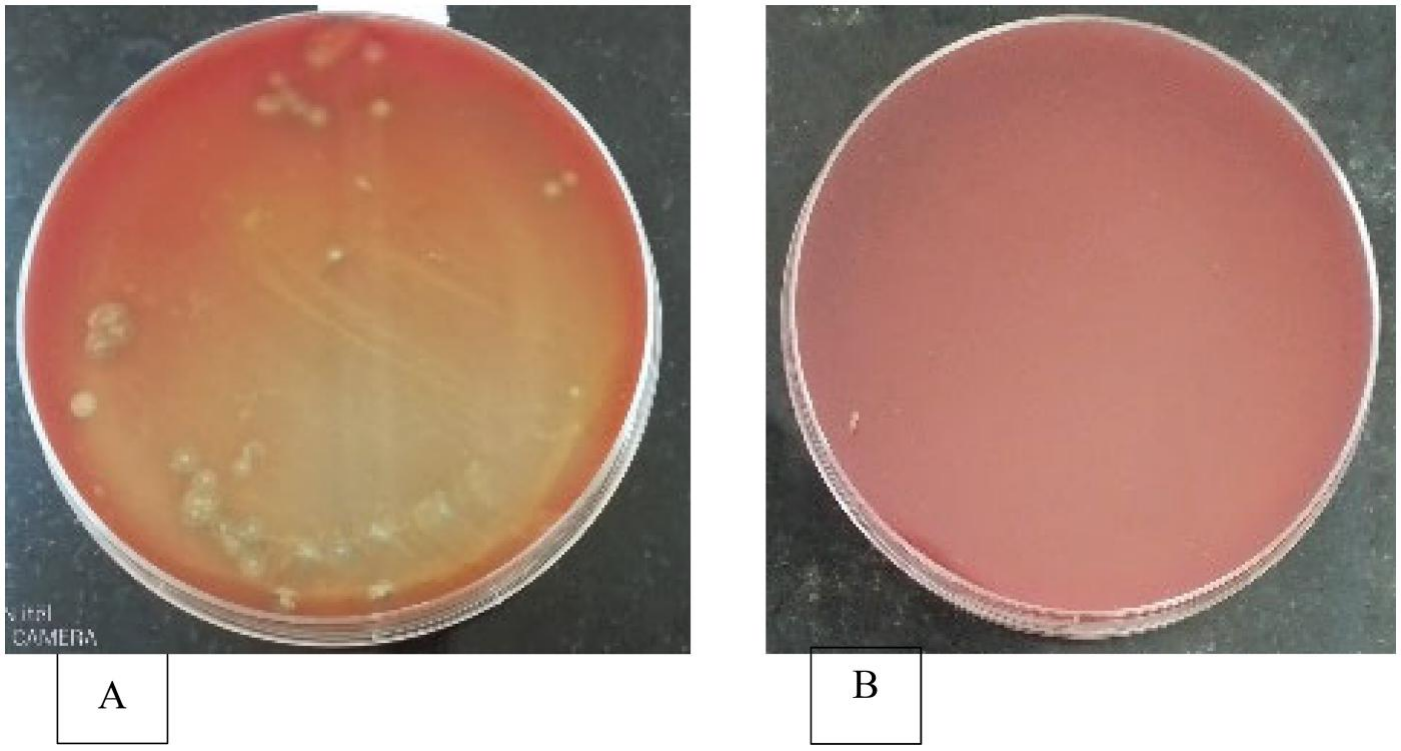
Isolation of potential biosurfactant producing microbes using hemolysis test.

Hemolytic activity was detected as the presence of clear zones around the bacterial colonies (Fig. 1). More microorganisms with greater clearance zones were found in the intestines, with small intestine derived samples having a wider clearance. The smallest clearance zone was seen in the gizzard and crop.

More colony forming units from the large intestines, as well as the small intestines, demonstrated hemolytic activity. However, small intestine derived samples had a larger clearance zone (5.6 mm) in the current study, followed by large intestines which was significantly different (p < 0.05) from those of the crop and gizzard (Table 2).

**Table 2.** Distribution of microbes showing clearance zones in sheep blood agar.

GIT region	Number of microbes showing clear zones	Average clearance zone (mm)
Crop	4	3.1^a^
Gizzard	7	2.6^a^
Small intestines	24	5.6^b^
Large intestines	32	4.7^b^
Grand mean		4.0
*p* Value		0.001

Rows with the same superscript are statistically insignificant while a different superscript means statistically different means at 5% significance level.

Colonies with larger clearance zones were subcultured to obtain pure cultures. These colonies had a variety of characteristics, with some exhibiting a slower development rate as indicated by a smaller colonized area (Table 3).

**Table 3.** Phenotypic characteristics of bacterial colonies subcultured from broiler birds’ GIT extracted samples.

GIT region	Description
Crop	Colonies were creamish in color and of fine texture They had smooth edges
Gizzard	Colonies were light-yellowish in color Edges were smooth
Small intestines	Colonies were clustered with some showing slow and rapid growth patterns Whitish color with fine structure was noted
Large intestines	Colonies showed reduced growth rate, Filamentous edges were noted Some were whitish in color with some creamish colors observed

#### Oil spreading method

**Figure 2.**
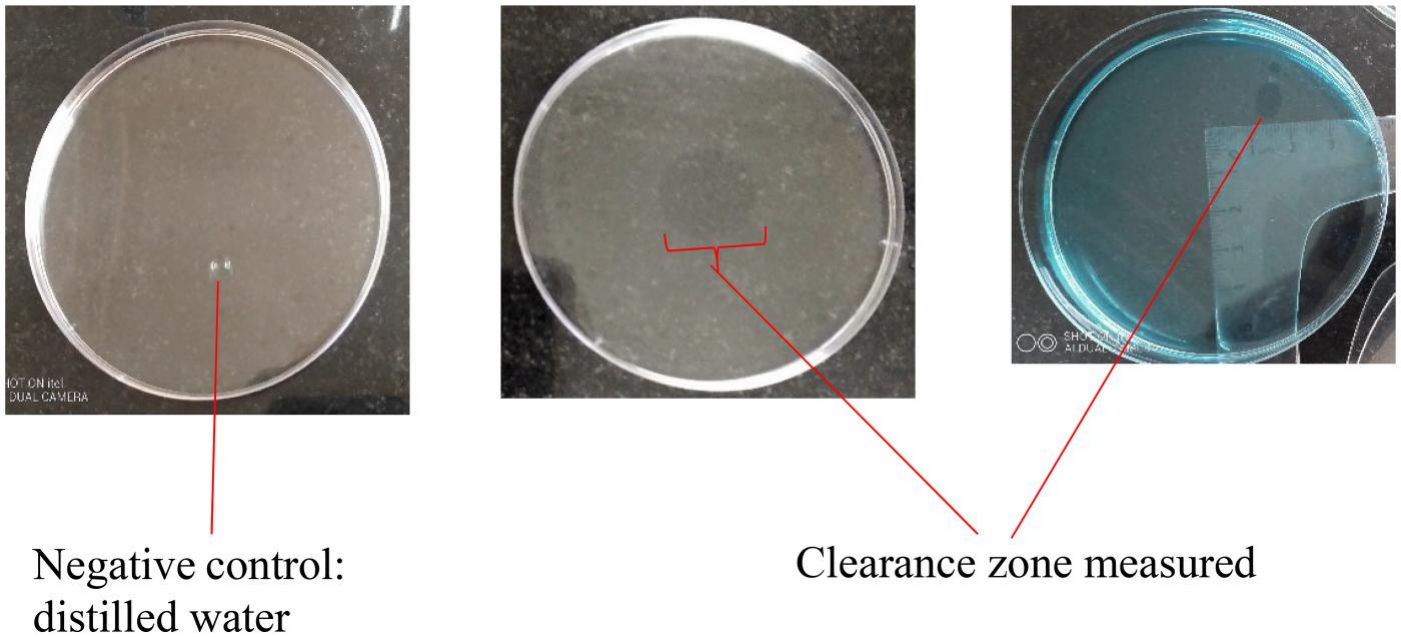
Appearance of clearance zones on oil spread technique.

Distilled water did not show any clearance activity unlike the biosurfactant containing supernatant (Fig. 2). The presence of biosurfactants in the supernatant will result in repulsion of the oil on water surface. This happens as a result of the amphiphilic properties of biosurfactants, thus a clearance activity confirms the availability of the biosurfactants in the supernatant.

With substantial clearance from small intestine samples, the gizzard did not demonstrate clearance zone (Table 4).

**Table 4.** Oil spread test clearance zones and average emulsification index (E_24_) for biosurfactants from different sections of broiler GIT.

GIT region	Average clearance zone (mm)	Av. E_24_ index (%)
Crop	6	26^a^
Gizzard	0	0
Small intestines	8.4	39^b^
Large intestines	6.5	26^a^
Grand mean	5.2	30.3
*p* Value	<0.05	<0.05

Rows with the same superscript are statistically insignificant while a different superscript means statistically different means at 5% significance level.

Clearance zones for oil spread technique were significantly different among all the tissues sampled. Larger clearing zones in intestine-derived samples may point to increased competition for resources among the occupants, as well as potentially powerful biosurfactants from this region.

#### Emulsification capacity (E_24_) of broiler GIT extracted biosurfactants

The extractions showed some emulsification capabilities proving useful as biosurfactants. On the E_24_ index, crop and large intestine derived samples trailed small intestine derived samples (Table 4).

#### Annotation by similarity using BLASTn

The DNA material was pure and of one type as indicated by a single strand on the gel^20^ thus the sample was good for sequencing. Similarity between the sequence queried and the biological sequences within the NCBI database was performed and a 98.29% identity was reviewed pointing to the *E. coli* strain 7.1994 as the microbe similar to the one producing the biosurfactants in the current study (https://doi.org/10.6084/m9.figshare.23574249.v1).

##### Genetic diversity of the biosurfactant secreting bacterial strains

**Figure 3.**
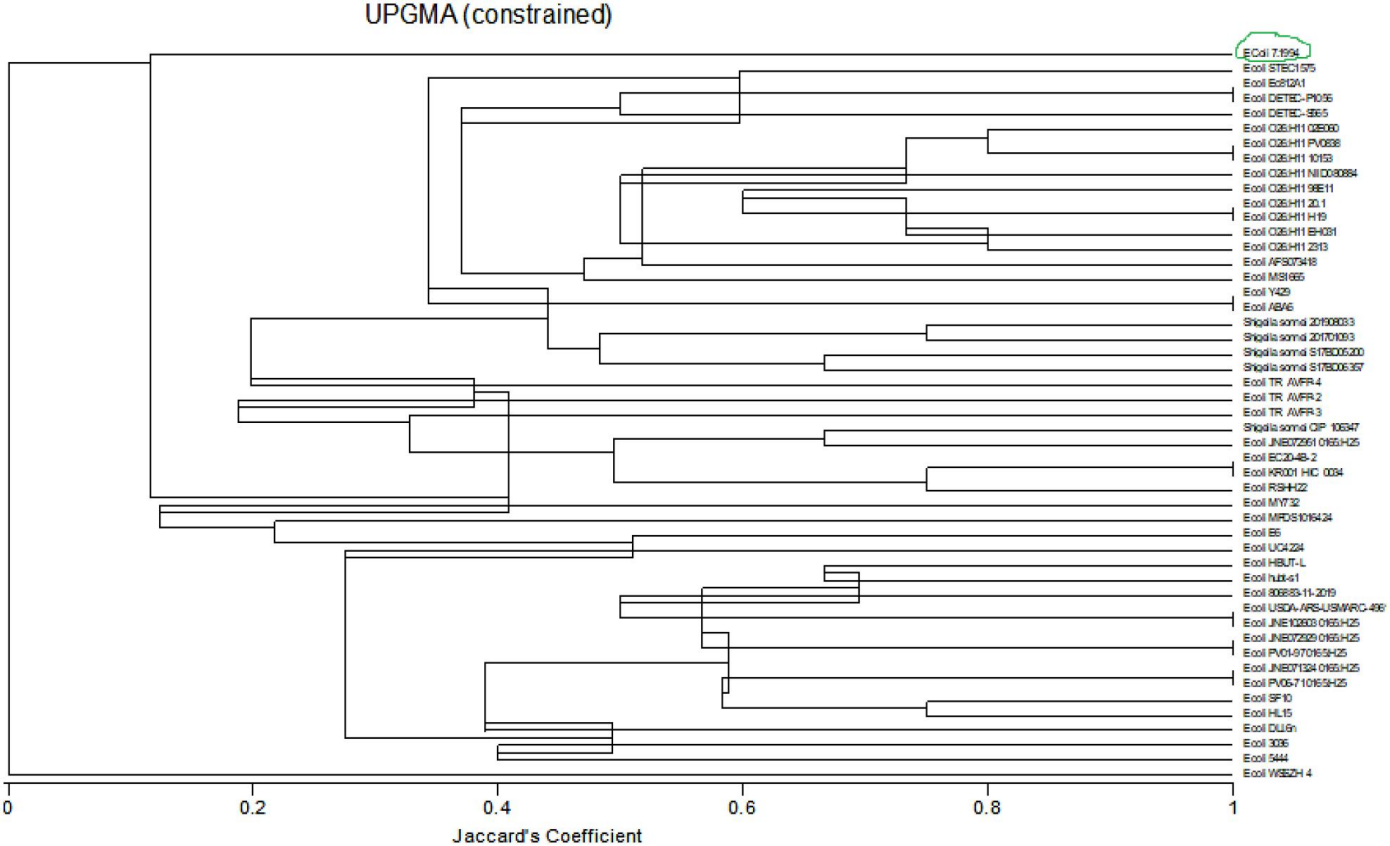
Dendogram of community relatedness of forty-nine bacterial strains based on the Jaccard’s coefficient.

A dendrogram (Fig. 3) was produced from phylogenetic analysis done using the Unweighted Pair Group Method (UPGM) of cluster analysis in MultiVariate Statistical Package (MVSP) version 3.22 Kovach Computing Services.

A total of forty-nine bacterial strains, consisting of 44 *E. coli* and 5 *Shigella sonnei* strains, which had a higher similarity score from BLASTn results were analyzed.

#### Gas chromatography mass spectrometry (GCMS)

The endogenous biosurfactants from *E. coli* strain 7.1994 related microbe showed the presence of Decane (C_10_H_22_), octadecane (C_18_H_38_), furane-2-carboxylate (C_11_H_7_FO_3_), and 1,7-Di-4-nitroheptan (C_21_H_23_NO_6_) phytochemical compounds with matching scores above 90% (Table 5).

**Table 5.** Major phytochemical compounds identified in biosurfactant GCMS analysis.

Compound name	Formula	Retention time	Area	Match score	CAS#
Decane	C_10_H_22_	5.672	1,394,666	90.6	124-18-5
DECANE	C_10_H_22_	5.672	1,256,257	90.9	124-18-5
Octadecane	C_18_H_38_	7.910	1,169,565	97.4	593-45-3
(4′-Fluorophenyl) furane-2-carboxylate	C_11_H_7_FO_3_	30.675	106,593	92.0	2000216-10-6
1,7-Di(2′-methoxyphenyl)-4-nitroheptan-1,7-dione	C_21_H_23_NO_6_	33.151	111,992	91.3	2000730-87-9

#### Fourier-transform infrared (FTIR) spectroscopy

**Figure 4.**
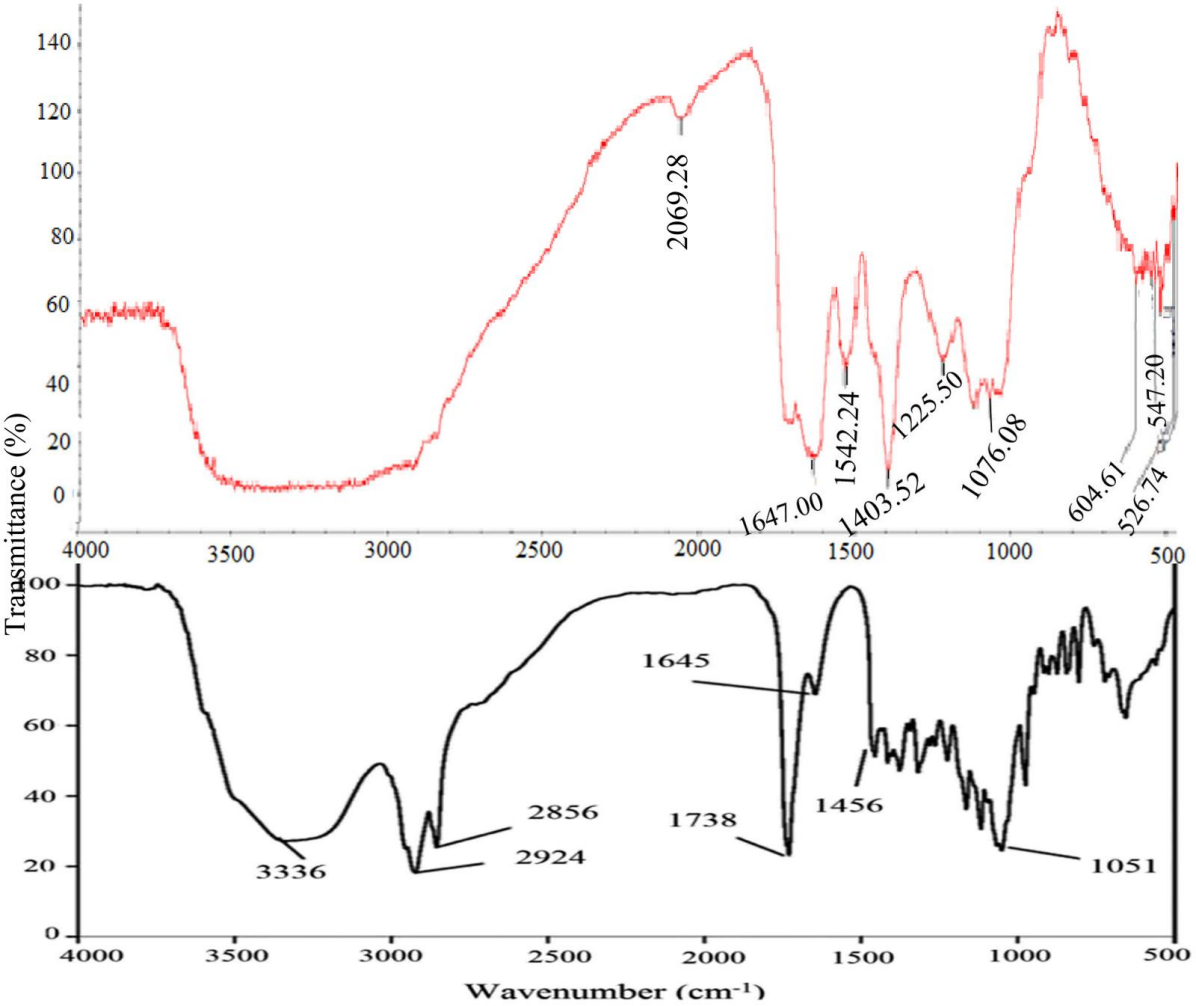
A comparison of FTIR spectroscopy of *Pseudomonas aeruginosa* (bottom) and crude endogenous biosurfactant (top) extracted from *E. coli* strain. *NB. Pseudomonas aeruginosa* adapted from Khademolhosseini et al.^21^

The extracted biosurfactants have peaks in the triple (2000–2500 cm^−1^), double (1500–2000 cm^−1^) and fingerprint regions (600–1500 cm^−1^) (Fig. 4).

Possible causes of observed peaks are presented in Table 6. A molecule’s ability to undergo the numerous reactions required to sustain life is provided by the carbonyl compound it contains, which occurred at 2069.28 wavenumber.

**Table 6.** FTIR peaks identified in Experimental biosurfactant.

No.	Wavenumber cm^−1^		Compounds responsible for the peak (Assignments)	Possible nutrient type
	Experiment	Literature		
1	2069.28	2100–1800	Carbonyl compound, transition metal carbonyls	carbohydrate
		2150–1990	Isothiocyanate (-NCS) representing nitrogen multiple and accumulated double bond compound	
2	1647.00	1680–1620	Olefinic (alkene) (Alkenyl C = C stretch),	Protein (Amide I)
		1650–1590/1650–1550	Ether and oxy compound (primary/secondary amine, NH bend)	
		1680–1630/1650–1600	Carbonyl compound (amide, quinone or conjugated ketone)	
		1690–1590	Nitrogen multiple and cumulated double bond compound (open-chain imino (–C = N–))	
3	1542.24	1560–1540/1555–1485	Nitrogen-oxy compound (aliphatic nitro compounds or aromatic nitro compounds)	Protein (Amide II)
4	1403.52	1420–1370	Sulfur-oxy compounds (organic sulfates)	Protein and collagen
		1420–1300	Carbonyl compound (carboxylate –carboxylic acid salt)	
		1410–1310	Alcohol and hydroxy compound (phenol or tertiary alcohol, OH bend)	
5	1225.50	1300–700	Saturated aliphatic (alkene/alkyl) (skeletal C-C vibrations)	Alkene
		1240–1190	Simple hetero-oxy compounds (phosphorus-oxy compounds –Aromatic phosphates (P-O-C stretch))	
6	1076.08	1150–1000	Aliphatic organohalogen compound (aliphatic fluoro compounds, C–F stretch)	Organic compound
		1150–1050/1140–1070	Ether and oxy compound (alkyl-substituted ether, C–O stretch or cyclic ethers, large rings, C–O stretch)	
		1090–1020	Primary amine, C–N stretch	
		1100–1000/1100–900	Common inorganic ions (phosphate ion or silicate ion)	
7	604.61	600–608	CH out of plane bending vibrations	Organic material

Synthesized from Nandiyanto et al.^29^

#### Screening biosurfactants for presence of antibiotics properties

Tetracyclines and sulfa drug properties in the extracted biosurfactant sample were found to be present (Table 7).

**Table 7.** Small intestine extracted biosurfactant sample radio receptor assay output.

Antibiotic test	Concentration (cpm)	Control point (cpm)
Tetracyclines^a^	274	1023
Beta lactams	1542	1036
Sulfa drugs^a^	189	1163
Amphenicols	584	406

^a^Positive result. Result reading less than control point result is positive whereas result reading greater than control point, result is negative.

Betalactams, which represent penicillin, produced negative results together with amphenicols. Tetracyclines and sulfa drugs were positive probably because of their continued use in broilers.

#### Antibacterial activity of broiler birds’ GIT extracted biosurfactants

The clearance zones were more noticeable in samples from the small intestine, with the positive control having the highest clearance (Table 8).

**Table 8.** Observed broiler GIT derived biosurfactants antimicrobial activity at 5% (m/v).

Biosurfactants’ sample source	Clearance effect
Crop	+
Small intestines	+++
Large intestines	++
Antibiotic (Terranox)	+++
Distilled water	–

Key: +: reduced growth of microbes; ++ growth reduction evident; +++ inhibition zone clearly visible; – no inhibition.

Similar to the positive control, the biosurfactants from the small intestine had distinct inhibition zones. For biosurfactants extracted from the small intestine, minimum inhibition activity was seen at a concentration of 5%.

## Discussion

The GIT microbial distribution map shows that even when birds are fed the same meal and exposed to the same environmental conditions, there were differences in the amount of microorganisms in their GIT. Choi et al.^22^ observed similar findings that were attributed to changes in time between feeding and sampling. In birds used in the current study, the large intestines contained more microbes than the small intestines. However, the intestines had less microbial population than the crop and gizzard, (*p* < 0.05). Each GIT section has distinct metabolic responsibilities that influence the microbial profile,^23^ hence varying microbial population densities were observed. However, same range populations in the crop and gizzard, as supported by Fathima et al.^6^ (p. 4), could be attributed to a shorter time period between feeding of the birds and sampling time. However, the intestines had less microbial populations which could be attributed to the acidic conditions of the proventriculus which can reduce populations of less acid adaptable microbes as the digesta passes through. More microorganisms in the large intestines may be credited to a stable environment because the digesta would have traveled through the preliminary GIT portions and some nutrients would have been absorbed. Also, because the quality of the digesta reaching the large intestines is partially uniform and rich in fibrous substance in the diet,^24^ the bacteria are not subjected to successive changes in the chemical composition of the digesta. The recent findings support Bailey’s^25^ arguments that the abundance and variety of the microbiota vary along the GIT, with fewer numbers of bacteria in locations with less acceptable circumstances and faster passage of gut contents. The gizzard has muscular walls and grit that serve primarily to physically crush the ingested feed. There were significant differences between birds in the crop microbial community. Some birds will pick on litter material regardless of whether they are fed *ad libitum* or not. This litter material may include a diverse microbial community, and because the crop will be the first port of call, variances in microbial communities amongst birds in the same area can exist.

Microbial variations observed in the current study in various parts of the same bird gut cements the findings of Oakley and Kogut,^26^ who proposed that location in the GIT, among other things, influences the composition of microbial populations. As diverse as they appear in the various portions of the GIT, it follows that a vast range of bacterial metabolites with various roles are released into the system of birds. If the crop and the gizzard’s narrower hemolytic clearing zones are any indication, they are related to their primary responsibilities, which do not necessitate a diverse array of activities. The crop moistens the feed in preparation for the physical activity of the gizzard. However, in the small intestines, there is a higher microbial activity,^25^ and other organisms such as *E. coli* live there. A greater clearance zone suggested that bacteria from this region were producing more biosurfactants.

The current clearance zone on oil spread is less than what was reported by Alkan et al.^17^ when they worked with lactic acid bacteria strains which ranged from 1.87 to 5.92 cm. Eighty percent of nutrient absorption occurs in the upper section of the small intestine,^27^ therefore, biosurfactants from this region are potential biofilm disruptors and beneficial metabolites because they are most likely produced by nutrient utilization related microorganisms. The oil drop collapse method did not produce results within the timed interval of 2 min. However, cultures gave a flat drop after the cut off time. This was partly attributed to a low concentration of biosurfactants in the extracts used, hence in the current study, this was not a reliable screening technique.

Some microorganisms have hemolysis characteristics but do not release biosurfactants outside of their cells.^28^ This will result in more microorganisms being reported on hemolysis activities, however on emulsification tests, the biosurfactants’ emulsification ability will be severely reduced because the test will be done by the biosurfactants rather than the microbes. Nayarisseri et al.,^29^ avers that samples with more than 30% emulsification activity indicates a greater activity. The emulsification values obtained in the current study were in the same range reported by Alkan et al.^17^ when they worked with lactic acid bacterial species which showed emulsification capabilities in the range 19.5–58%. The tests performed in the current study show that the small intestine derived sample has higher activity. The findings augment assertions by Sambanthamoorthy et al.,^30^ that biosurfactants have important qualities, among them substantial emulsification activity. Small intestine samples showed an upper hand and were examined further for their antibacterial qualities to see if they can suppress the growth and development of common infections under stressful settings.

The phylogenetic dendrogram shows the genetic evolutionary relationships or similarity proportion between the identified bacterial strains.^31^ The dendrogram shows two major groups, *E. coli* strain WSSZH4 and the other strains emanating from the other group. This points to the fact that *E. coli* strain WSSZH4 is distinct from all the other forty-eight strains studied in this dendrogram. This is supported by Devanga Ragupathi et al.^32^ who affirms that there are some distinct strains of *E. coli* which are not related to other strains of *E. coli* or *Shigella* strains. *Shigella* is a genus of Gram-negative bacteria genetically closely related to *E. coli.*^33^ Some of its characteristics include it being a facultative anaerobic, non-spore-forming, nonmotile, and rod-shaped bacterium.^34^ The dendrogram shows that the *Shigella* strains are closely related to some strains of *E. coli*, particularly *E. coli* strain STEC 1575 and *E. coli* strain JNE072951 0165:H25. Since the *E. coli* and *Shigella* can occur together in the gut of broilers and have a common score in this study, there is a chance that these microbes exchanged their genetic material between the involved strains, equipping the *E. coli* strain with the biosurfactant production genes. Shad and Shad^35^ have reported the ability of cross-immunisation by some other strains of *Shigella* in the gut. This partly explains the biosurfactant production ability of the *E. coli* strain in the current study. The dendrogram affirms the BLASTn analysis output as indicated by an *E. coli* strain 7.1994 (green encircled on Fig. 3 dendrogram) with a Jaccard’s coefficient less than 0.2, meaning less than 20% similarity with the rest of the other strains picked in the BLASTn analysis. The current findings point to an *E. coli* bacterium closely related to *E. coli* strain 7.1994/NIST 0056 bacterium being responsible for biosurfactant production in the gut of broilers.

A GCMS match factor score above 90 is considered an excellent match, 90 is a good match, 70–80 is a fair match, and 60% is a poor match.^36^ As a result, the biosurfactant compounds discovered in the current study fall into the excellent category, with Octadecane receiving a highest score of 97.4% match factor. Octadecane, a hydrophobic molecule, is a straight chain alkane carrying 18 carbon atoms. It has a role as a bacterial metabolite and a plant metabolite.^37^ Decane (CAS# 124-18-5) is an aliphatic hydrocarbon primarily derived from crude oil. In addition to the rubber and paper industries, it is used as a solvent in organic syntheses. Furane-2-carboxylate (4′-Fluorophenyl) has been reported as a factor with potential inhibitory activity against bacterial swarming^1^ and likely inhibit extracellular polysaccharide production.^38^ The inhibitory activity seen in the current study may be due to trace amounts of this compound, which would give the secreting microbe immunity. This is a positive development for the creation of a novel anti-infective strategy to reduce the overuse of synthetic drugs in broiler production, as supported by Rütschlin and Böttcher.^39^ 1,7-Di(2′-methoxyphenyl)-4-nitroheptan-1,7-dione may be associated with control of inflammation, wound and muscular atrophy, and immune disorders as put forward by Shih et al..^40^ This chemical may be useful in microbe defence mechanisms against certain drugs.

Other biosurfactants have been reported to have peaks in the regions noted in the current study.^41^^,^^42^ However, no peaks were observed above the 2100 wavenumbers (cm^−1^). Regarding the number of peaks, there are seven peaks, informing that the analyzed biosurfactant is a small organic component as supported by Nandiyanto et al.^43^ There is evidence of functional groups in the extracted biosurfactants as evidenced by absorbance bands above 1500 cm^−1^ (Fig. 4). Since there were no peaks in the 3650–3200 wavenumber range, the sample had dried completely and had not absorbed any chemotherapeutic water.^43^ A carbonyl compound gives a biological molecule the ability to generate new molecules and be altered with a variety of other functional groups. Because they are polar in nature, carbonyl compounds have minute positive and negative charges.

The hydrophilic and hydrophobic regions of chemically produced surfactants are clearly separated into a hydrophilic head group (charged or polar) and a hydrophobic tail, which is made up of linear alkyl groups.^44^ The observed peak indicates the presence of amides or carboxylates functional groups in the double bond region because it occurred below 1700 cm^−1.^^43^ Peaks in the range of 1670–1620 cm^−1^, as highlighted by Nandiyanto et al.,^43^ represent unsaturation bonds (double and triple bond). As a result, an unsaturated bond accounts for the observed peak at 1647 wavenumbers. It is possible that the benzene ring, which produced a peak at wavenumber 1542, is attributable to this alkene stretch. This nitrogen-oxy compound supports the discovery of the aliphatic hydrocarbon decane (CAS 124-18-5) made by the GCMS. The biosurfactant possesses polar properties and some antibiotic-like ionizable compounds (at 1076.08 wavenumbers).^45^

Tetracyclines and sulfonamides, picked by the radio receptor assay, are two antibiotic classes of popular veterinary use for animals.^2^^,^^45^ Therefore, it stands to reason that some intestinal microbes may have evolved specific mechanisms to produce similar compounds for their defence. However, beta lactams (penicillins) are the preferred medication for humans,^46^ so their use in animals is constrained. This partially explains the low concentrations of these substances because microbes lack the ability to adjust to and become accustomed to the synthesis of such substances. Biosurfactants have tremendous therapeutic potential and antimicrobial qualities, and they can accomplish their intended functions with fewer side effects. The current study results support the hypothesis put forth by Sambanthamoorthy et al.^30^ that biosurfactants had stronger antibacterial properties. Growth inhibition zones were observed around the biosurfactant extracts, but not around the negative control (distilled water). On samples from the crop and large intestines, antibacterial activity was, however, barely detectable. *Staphylococcus aureus* responded more to biosurfactants addition. The results of the study support the claims made by Naughton et al.^47^ that biosurfactants can have a number of functions, including antibacterial activity. The negative control (distilled water) had the greatest number of colony forming units as it failed to suppress microbial growth. *Escherichia coli* exhibited some resistance to the crop’s biosurfactants. However, there was significant clearance around the biosurfactants retrieved from the intestines.

### Conclusion and recommendations

A biosurfactant-secreting microbe was isolated from broiler GIT and identified as *E. coli* strain 7.1994. The potential use of the biosurfactants from this strain of *E. coli* in antimicrobial resistance challenge lies in its exhibited oil spreading activity and a higher emulsification index, 39%, in vegetable oil. The FTIR and GCMS spectra show that the biosurfactant is a glycolipid, derivative of decane without hydrate component but with double and triple bonds related to ketones. Therefore, this *E. coli* strain 7.1994 and its biosurfactant could be suitable for use in the fight against antimicrobial resistance challenge from broiler products. Researchers recommend further studies on optimization of biosurfactant release and *in-vivo* trials with broiler chickens.

## Data Availability

Data are presented in-text, and a link has been provided for data in repository.

## Footnotes

1 Swarming is a coordinated form of motility in which bacteria can migrate in groups of flagella-driven cells over semi-solid surfaces (46).
